# Factors Associated with Nutritional Deficiency Biomarkers in Candidates for Bariatric Surgery: A Cross-Sectional Study in a Peruvian High-Resolution Clinic

**DOI:** 10.3390/nu14010082

**Published:** 2021-12-25

**Authors:** Adrian Riva-Moscoso, Raisa N. Martinez-Rivera, Gianfranco Cotrina-Susanibar, Fortunato S. Príncipe-Meneses, Diego Urrunaga-Pastor, Gustavo Salinas-Sedo, Carlos J. Toro-Huamanchumo

**Affiliations:** 1 Escuela de Medicina, Universidad Peruana de Ciencias Aplicadas, Lima 15067, Peru; u201612662@upc.edu.pe (A.R.-M.); u201613482@upc.edu.pe (F.S.P.-M.); 2 Facultad de Ciencias de la salud, Escuela Profesional de Medicina Humana, Universidad Nacional de Piura, Piura 20002, Peru; 0902016031@alumnos.unp.edu.pe; 3 Escuela de Medicina Humana, Universidad Católica Santo Toribio de Mogrovejo, Chiclayo 14012, Peru; 72114110@usat.edu.pe; 4 Facultad de Ciencias de la Salud, Universidad Científica del Sur, Carrera de Medicina Humana, Lima 15067, Peru; 5 Unidad de Investigación Multidisciplinaria, Clínica Avendaño, Lima 15074, Peru; gsalinas@clinicaavendanoperu.com; 6 Unidad para la Generación y Síntesis de Evidencias en Salud, Universidad San Ignacio de Loyola, Lima 15012, Peru

**Keywords:** obesity, bariatric surgery, nutrition, nutritional assessment

## Abstract

Previous studies have described multiple nutritional deficiencies after bariatric surgery (BS). However, few studies have evaluated these deficiencies prior to BS, specifically in Latin America. This study aimed to determine the factors associated with nutritional deficiency biomarkers in candidates for BS in Peru. We included adults of both sexes, aged 18 to 59 years, admitted to a Peruvian clinic with a body mass index (BMI) ≥30 kg/m^2^; they were candidates for BS from 2017 to 2020. We considered the serum levels of hemoglobin and albumin (in tertiles) as the nutritional deficiency biomarkers. In order to assess the associated factors, we calculated crude (cPR) and adjusted prevalence ratios (aPR) with their respective 95% confidence intervals (95%CI). We analyzed 255 patients: 63.1% were males, with a mean age of 37.1 ± 10.3 years and mean hemoglobin and albumin values of 14.0 ± 1.5 g/dL and 4.6 ± 0.4 g/dL, respectively. We found that males (aPR = 1.86; 95%CI: 1.26–2.73; *p* = 0.002), participants between 30 and 49 (aPR = 2.02; 95%CI: 1.24–3.28; *p* = 0.004) or 50 years or more (aPR = 2.42; 95%CI: 1.35–4.35; *p* = 0.003), participants with a BMI ≥40 kg/m^2^ (aPR = 1.68; 95%CI: 1.09–2.60; *p* = 0.018), participants with impaired high-density lipoprotein levels (aPR = 1.43; 95%CI: 1.01–2.05; *p* = 0.049) and individuals in the high tertile of C-reactive protein (aPR = 6.94; 95%CI: 3.37–14.32; *p* < 0.003) had a higher probability of being in the lower tertile of albumin. In addition, we found that the male sex (aPR = 6.94; 95%CI: 3.37–14.32; *p* < 0.001) and elevated cholesterol levels (aPR = 0.71; 95%CI: 0.52–0.97; *p* = 0.034) were associated with the lowest hemoglobin tertile. In our setting, nutritional deficiency biomarkers were associated with sociodemographic, anthropometric and laboratory markers. The pre-bariatric surgery correction of nutritional deficiencies is essential, and can prevent major complications after surgery.

## 1. Introduction

The prevalence of obesity tripled between 1975 and 2016. In 2016, it was estimated that 39% of adults worldwide had overweight, and 13% had obesity [1]. Obesity represents a public health problem due to its association with diseases such as high blood pressure, type 2 diabetes mellitus, cardiovascular diseases, and cancer [2]. Over the years, obesity has ceased to represent a problem only in people at a high socioeconomic level [2], as the burden of obesity has been increasing, and is unevenly distributed in all socioeconomic groups. Among individuals with low resources, obesity is associated with a greater number of comorbidities compared to those with medium or high incomes, further driving health inequities, and requiring the development of national public policies to prevent and treat obesity-related disease [3].

Bariatric surgery is currently the best alternative to combat morbid obesity, providing better control of chronic diseases, a considerable decrease in weight, and long-term weight-loss maintenance, improving quality of life and decreasing mortality [4]. Furthermore, the demand for this procedure has increased in recent years. In 2016, it was estimated that 685,874 bariatric surgery interventions were carried out worldwide [5]. Likewise, according to the latest data, in 2017 a total of 228,000 bariatric procedures were reported in the United States [6]. Despite the benefits of these interventions [4], as with any procedure, complications can occur, some of which are related to the procedure, such as gallstones, marginal ulceration, internal hernia and intussusception [7]. Likewise, the anatomical and physiological alteration of the gastrointestinal tract produced by surgery makes the patient more susceptible to disabling diseases such as anemia, protein malnutrition, deficiencies of calcium and vitamin D micronutrients, low serum levels of other fat-soluble vitamins, and essential mineral deficiencies including magnesium, zinc, copper, and selenium [8].

Previous studies carried out in United States and Europe [9,10,11,12,13] evaluated nutritional deficiencies in patients after undergoing bariatric surgery, with an average post-surgery follow-up ranging from 12 to 24 months. On the other hand, nutritional deficiencies have been explored less frequently in patients who are candidates for bariatric surgery [14,15,16,17,18], and even less so in Latin America [19]. It is relevant to obtain information related to nutritional deficiency biomarkers prior to bariatric surgery, and to identify the associated factors in order to explore the conditions that could improve later outcomes, given the greater influx of patients who are interested in undergoing bariatric surgery in Peru [20]. In addition, the evidence generated could serve as the basis to better understand the baseline status of patients undergoing bariatric surgery, to provide better preparation and medical care before and after surgery, and thus to prevent [14] the consequences of these deficiencies in the long term, which can lead to more serious scenarios for the patient.

Therefore, our study aimed to explore these deficiencies in patients who were candidates for bariatric surgery at a high-resolution private clinic in Lima, Peru.

## 2. Materials and Methods

### 2.1. Study Design and Context

We performed a secondary analysis of the database for epidemiological surveillance purposes created by a private clinic in Lima, Peru. This private clinic is a high-resolution center specialized in the treatment of obesity and metabolic and nutritional diseases. This center performed in 2021 more than 700 bariatric surgeries, including sleeve gastrectomies and gastric bypass.

### 2.2. Study Population

We included adults of both sexes between 18 and 59 years old with a body mass index (BMI) greater than or equal to 30 kg/m^2^ who attended the private clinic and qualified as candidates for a surgical procedure as treatment for obesity from 2017 to 2020. We excluded pregnant women, participants self-reporting harmful alcohol intake, a history of acute myocardial infarction or findings consistent with myocardial ischemia according to an electrocardiogram, a history of viral hepatitis (hepatitis B and C), autoimmune liver diseases, liver deposition diseases (hemochromatosis, hemosiderosis, and the chronic use of drugs that can induce secondary non-alcoholic steatohepatitis: amiodarone, tamoxifen, methotrexate, corticosteroids, valproate and nitrofurantoin), and patients with a liver biopsy with less than 10 portal spaces and/or a depth less than 5 mm. They were excluded because this could represent potential confounders, according to the literature.

### 2.3. Outcomes

We considered hemoglobin and albumin as nutritional deficiency biomarkers. Similarly, to previous studies, these laboratory markers were analyzed as tertiles [21,22]. The hemoglobin sample was collected in tubes containing ethylenediaminetetraacetic acid for complete blood count tests, while the bromocresol green colorimetric assay was used for albumin.

### 2.4. Independent Variables

#### 2.4.1. Sociodemographic Characteristics

We considered the following sociodemographic variables: sex (female, male) and age (18 to 29 years, 30 to 49, and 50 and over).

#### 2.4.2. Medical and Personal History

We included comorbidities including hypertension (no, yes), type 2 diabetes mellitus (no, yes) and hypothyroidism (no, yes). In addition, we assessed smoking habits (no, yes) in the participants through self-reporting.

#### 2.4.3. Laboratory and Anthropometric Markers

We evaluated the lipid profile in the participants, including cholesterol (defined as being elevated at a value greater than or equal to 200 mg/dL), triglycerides (defined as being elevated at a value greater than or equal to 150 mg/dL), high-density lipoprotein (HDL) (defined as being altered with a value less than 40 mg/dL in men, or less than 50 mg/dL in women), and low-density lipoprotein (LDL) (defined as being altered with a value greater than or equal to 130 mg/dL). We calculated the Homeostatic Model Assessment for Insulin Resistance (HOMA-IR) in mg/dL using the following formula: (glucose × insulin)/405. The body mass index (BMI) was calculated using the following formula: weight (kg)/[height (m)]^2^; it was then categorized as 30–34.99 kg/m^2^, 35–39.99, or 40 or more. The C-reactive protein values were considered altered with values greater than 10 mg/L. Nonalcoholic fatty liver disease (NAFLD) was diagnosed by the histopathological examination of liver biopsy taken during surgery. All of the liver biopsies were reviewed by two experienced pathologists.

### 2.5. Statistical Analysis

We performed the statistical analysis using the statistical package STATA v14 (StataCorp, College Station, TX, USA). The descriptive analysis of the quantitative variables was presented using measures of central tendency and dispersion, as appropriate, after evaluating their normality. The variables with a normal distribution were presented as means and standard deviation, and those with a non-normal distribution were expressed as a median and interquartile range (IQR). On the other hand, the qualitative variables were described using relative and absolute frequencies.

The bivariate analyses of the tertiles of the nutritional deficiency biomarkers and the qualitative covariates of interest were performed using the Chi-square test or Fisher’s exact test, depending on the expected values. Likewise, we used the ANOVA or Kruskal Wallis test to compare the numerical variables among the tertiles of the outcome variables. We constructed generalized linear models of the Poisson family with logarithmic link function and robust variances to evaluate the factors associated with the nutritional deficiency biomarkers in the study sample. For the multivariable analysis, the tertiles of the nutritional deficiency biomarkers were dichotomized, and the association measure was calculated for the tertile of greater clinical relevance. The forward manual selection method was used to select the factors associated with the outcome variables. We calculated the crude (cPR) and adjusted (aPR) prevalence ratios with their respective 95% confidence intervals (95%CI). A *p* value <0.05 was considered statistically significant.

We decided not to stratify by sex, as we tested for interaction with the log-likelihood ratio test, and obtained a *p*-value > 0.05.

### 2.6. Ethics

The present study was approved by the Institutional Review Board of the Clínica Avendaño (010–2021-CIEI). Participant consent was not required, and the study database was anonymized.

## 3. Results

### 3.1. Characteristics of the Study Sample

We included 255 patients with a mean age of 37.1 ± 10.3 years who were candidates for bariatric surgery. We found that 63.1% (*n* = 161) were male, and only 12.9% (*n* = 33) were 50 years or older. Furthermore, 18.4% (*n* = 47) had hypertension, while 6.3% (*n* = 16) and 11.0% (*n* = 28) had type 2 diabetes mellitus and hypothyroidism, respectively. Furthermore, in relation to the lipid profile, 42.3% (*n* = 108) had elevated cholesterol levels, and 58.4% (*n* = 149) had altered HDL values. We found that the mean HOMA-IR was 6.4 ± 4.5 units; 96.5% (*n* = 246) of patients had NAFLD, 72.1% (*n* = 184) had a BMI greater than or equal to 35 kg/m^2^, and the median CRP was 6.1 units (IQR: 3.1–10.2) (Table 1). On the other hand, the mean hemoglobin and albumin values were 14.0 ± 1.5 and 4.6 ± 0.4, respectively (Table 2).

### 3.2. Bivariate Analysis According to the Tertiles of Albumin and Hemoglobin

We found differences with HDL, NAFLD, BMI categories and CRP tertiles with respect to the albumin tertiles. Likewise, we found differences between sex, hypothyroidism, and triglyceride and HDL values compared to the tertiles of hemoglobin. Furthermore, we found a higher median HOMA-IR in the participants in the upper hemoglobin tertile compared to the other two tertiles (6.2 vs. 5.6 vs. 4.5; *p* < 0.001). Table 3 shows the results of the bivariate analysis between the covariates and the outcomes of interest.

### 3.3. Factors Associated with Nutritional Deficiency Biomarkers in the Study Sample

We found that men (aPR = 1.86; 95%CI: 1.26–2.73; *p* = 0.002), participants with an age between 30 and 49 (aPR = 2.02; 95%CI: 1.24–3.28; *p* = 0.004) and those aged 50 years or more (aPR = 2.42; 95%CI: 1.35–4.35; *p* = 0.003) were more likely to be in the lower tertile of albumin. In addition, the participants with a BMI of 40 kg/m^2^ or more (aPR = 1.68; 95%CI: 1.09–2.60; *p* = 0.018), with altered HDL levels (aPR = 1.43; 95%CI: 1.01–2.05; *p* = 0.049) and high CRP values (aPR = 1.83; 95%CI: 1.13–2.96; *p* = 0.013) were associated with a higher prevalence of being in the lower albumin tertile. Men (aPR = 6.94; 95%CI: 3.37–14.32; *p* < 0.001) had a greater probability of being in the lower hemoglobin tertile, while individuals with high cholesterol levels (aPR = 0.71; 95%CI: 0.52–0.97; *p* = 0.034) had a lower prevalence (Table 4).

## 4. Discussion

### 4.1. Main Findings

In this study of 255 adult candidates for bariatric surgery, several factors were found to be associated with nutritional deficiency biomarkers. Male sex, ages of over 30 years, a BMI greater than or equal to 40 kg/m^2^, altered HDL levels, and being in the high-CRP tertile were associated with having low albumin levels. In addition, males were associated with a higher probability of having low hemoglobin levels, while a higher cholesterol level was associated with a lower probability of having low hemoglobin levels. Although the factors associated with nutritional deficiency have been studied, most were explored in patients after they had undergone bariatric surgery [23,24,25,26,27]. To our knowledge, this is one of the first studies carried out in a Latin American population, and it provides novel and interesting information on the subject.

### 4.2. Comparison with Previous Studies

Although most previous studies have evaluated the determinants of nutritional deficiency in patients following bariatric surgery, in the last decade, there has been greater interest in the evaluation of nutritional deficiency in patients who are candidates for bariatric surgery [15,16,18,19,28]. However, most of these studies were carried out in European [15,16], Asian [17] and North American [18] populations.

In a study carried out in Spain [16] in 115 female patients who were candidates for bariatric surgery, it was found that 6.1% had hypoalbuminemia and 2.6% had hemoglobin values less than 12 g/dL. In addition, another study carried out in Germany, in patients with morbid obesity [15], found that 88% had low serum albumin results (<40 g/dL), describing an association between morbid obesity and a low consumption of essential micronutrients. On the other hand, a study carried out in South Korea [17] evaluated 215 patients who were candidates for bariatric surgery, and reported mean hemoglobin values for both sexes of 14.5 g/dL; however, the prevalence of anemia differed between men and women, with 1.3% and 7.4%, respectively.

Most of the studies conducted in Latin America evaluated nutritional deficiencies after bariatric surgery [29]. However, a previous study carried out in Chile [19] evaluated nutritional deficiencies in 103 women, including the measurement of albumin, hemoglobin, ferritin, and vitamins and minerals such as selenium and zinc. In addition, it included measurements such as dual-energy X-ray absorptiometry (DEXA) and eating habits. Our study included people of both sexes, and the sample was larger. Indeed, our study provides relevant information on the Peruvian population.

### 4.3. Interpretation of the Results

We found that being over 30 years of age was associated with a higher probability of being in the lower tertile of albumin. Preoperative hypoalbuminemia has been identified as a modifiable factor which is predictive of serious complications in bariatric surgery candidates [30]. An inverse relationship has been described between age and serum albumin, and we more frequently find older adult patients with hypoalbuminemia [31]. In a cohort in which the participants had a mean age of 34.8 years, the mean albumin values were 4.1 g/dL one year after the intervention, and 4.3 g/dL after 3 years [32]. Similarly, other studies have shown that age is not a cause of hypoalbuminemia; rather, this marker is a risk factor of mortality [33]. The inflammatory state and, particularly, high interleukin-6 and tumor necrosis factor (TNF)-alpha concentrations are the two main markers associated with hypoalbuminemia [33].

Likewise, we found that having altered HDL values was associated with a greater probability of being in the lower albumin tertile. To our knowledge, no previous study has described a similar association in patients who are candidates for bariatric surgery. This finding could be important for two reasons. First, a previous study found that the HDL values in candidates for bariatric surgery improved after surgery, suggesting its benefit [34]. As such, this variable could be useful as a potential marker of a successful metabolic status after surgery. Second, because HDL was associated with the lower albumin tertile, this lipid marker could be important to predict hypoalbuminemia in candidates, and therefore these patients could be considered to receive nutritional support.

We found that a BMI greater than or equal to 40 kg/m^2^ was associated with a greater probability of having low albumin values. In a study conducted in China, the prevalence of hypoalbuminemia in candidates for bariatric surgery was 11.8% [35]. These authors also found a statistically significant association between elevated BMI levels and serum albumin deficiency. The patients with a BMI of 40–45 and >45 kg/m^2^ had a prevalence of hypoalbuminemia of 12.8% and 30.3%, respectively [35]. One possible explanation for this finding is the difference in races or complex mechanisms not yet identified between BMI and albumin values. Another possible explanation could be the state of chronic inflammation represented by obesity, in which adipocyte hypertrophy and hypoxia lead to the production of pro-inflammatory cytokines, such as TNF-alpha. It is plausible that this state of inflammation could lead to altered serum albumin levels among patients with obesity [36].

On the other hand, we found an association between the high tertile of CRP and low albumin values in the participants of the study sample. This finding is consistent with that described in Italy, in which candidates for bariatric surgery with low levels of albumin were more likely to be in the group with high levels of CRP (with a mean of 7.7 mg/L). This is generally due to the chronic pro-inflammatory state of patients with obesity, which results in an increase in acute-phase proteins such as CRP, as well as a decrease in albumin concentrations [37], both of which are clinical markers of inflammation. The finding of hypoalbuminemia should be interpreted with caution because, in inflammatory stages, albumin tends to decrease, and thereby overlap its true value [37].

In our study, male sex was associated with lower hemoglobin levels. A previous study showed that the high prevalence of anemia could be due to the lack of access to iron-rich foods and a lack of adherence to oral iron treatment due to its adverse gastrointestinal effects. However, the highest prevalence of anemia was found in females (21.6%), most likely due to the large number of women of reproductive age included in the study [14]; this result was also evidenced in another similar study carried out in South Korea [17]. On the other hand, a high cholesterol level was associated with low hemoglobin levels in the study sample. In a retrospective study analyzing the nutritional profile of patients who had undergone bariatric surgery in Brazil [38], the preoperative prevalence of low hemoglobin levels was 6.5%, while the prevalence of altered cholesterol levels was 54.7%. It should be noted that in the follow-up 7 to 10 years after undergoing bariatric surgery, the number of patients with hypercholesterolemia in Chile [39] reduced by 88%, while the prevalence of anemia in these patients remained at 31%. However, this association may not be clinically relevant, and could be due to a spurious association. Therefore, it has to be corroborated by future studies.

### 4.4. Clinical Practice Relevance

Most studies focus on post-bariatric surgery results, while we reported the results of patients who were candidates for bariatric surgery. These findings could help to avoid major complications after surgery, with timely actions in the highest risk group. The role of albumin as a nutritional biomarker has been described previously, specifically in patients undergoing abdominal surgery [40]. Likewise, the evaluation of hemoglobin as a marker of malnutrition was proposed in a previous study. Additionally, the authors concluded that its evaluation is even more useful in older adults [41].

The correction of low hemoglobin values is a challenge which can lead to a reduction in future complications, with previous studies reporting the development of anemia in around 15% of post-bariatric surgery patients. Likewise, 20% of these individuals fail to recover after 1 year of follow-up [42]. Moreover, in this latter study, women presented the lowest hemoglobin levels, probably due to blood loss during the menstrual cycle.

Furthermore, serum albumin levels can be used as a prognostic tool to help predict the medical and surgical outcomes of the patient. The association of low albumin levels with adverse patient outcomes is due to the fact that albumin is a biomarker of severe protein malnutrition. Likewise, it has been reported that the preoperative detection of hypoalbuminemia was associated with adverse outcomes, specifically delayed wound healing, poor surgical outcomes, the need for repeated surgery, and higher readmission rates [36]. Therefore, the consumption of foods rich in protein (eggs, fish, meat, soy products, and legumes) and the reduction of carbohydrate and fat intake is recommended to avoid these complications [43].

### 4.5. Limitations and Strengths

This study has some limitations. First, the cross-sectional design did not allow us to establish causality among the associated factors and the nutritional deficiency biomarkers in the study sample. Second, because it was an analysis of a secondary database, it was not possible to include minerals and other vitamins that could be relevant as nutritional deficiency biomarkers according to previous studies. Despite these limitations, to our knowledge, this study represents the first in Peru and one of the first in Latin America to evaluate the factors associated with nutritional deficiency biomarkers in patients who are candidates for bariatric surgery. According to the literature, this group is of special interest because although the nutritional deficit could be greater after surgery, if the patient already has this condition before surgery, it could be aggravated after the intervention. As such, it is important to act in a timely manner to avoid greater consequences. In addition, compared to developed countries, the Peruvian population is probably at greater risk [18]. The results allow the identification of modifiable and non-modifiable markers for the development of pre-surgical strategies that can be implemented to avoid adverse outcomes after surgery.

## 5. Conclusions

Nutritional deficiency biomarkers were associated with sociodemographic, anthropometric and laboratory factors in patients who were candidates for bariatric surgery in Peru. This deficit can increase the risk of adverse outcomes and complications after bariatric surgery. Pre-surgical interventions aimed at reducing nutritional complications following surgery are needed in patients undergoing bariatric surgery.

## Figures and Tables

**Table 1:** Demographic characteristics, medical history, and laboratory and clinical markers of the study sample (*n* = 255).

Characteristics	*n* (%)
**Sex**	
Female	94 (36.9%)
Male	161 (63.1%)
**Age**	37.1 ± 10.3 *
18 to 29 years	66 (25.9)
30 to 49	156 (61.2)
50 or over	33 (12.9)
**Hypertension**	
No	208 (81.6%)
Yes	47 (18.4%)
**Type 2 diabetes mellitus**	
No	239 (93.7%)
Yes	16 (6.3%)
**Hypothyroidism**	
No	227 (89.0%)
Yes	28 (11.0%)
**Smoking habit**	
No	195 (76.5%)
Yes	60 (23.5%)
**Cholesterol**	194.2 ± 38.8 *
Normal	147 (57.7)
Elevated	108 (42.3)
**Triglycerides**	151 (112–207) **
Normal	131 (51.4)
Elevated	124 (48.6)
**HDL**	43.8 ± 11.5 *
Normal	106 (41.6)
Altered	149 (58.4)
**LDL**	116.2 ± 33.8 *
Normal	90 (35.3)
Altered	165 (64.7)
**CRP**	6.1 (3.1–10.2) *
Low tertile	2.5 (1.8–3.1) **
Intermediate tertile	6.2 (5.0–7.4) **
High tertile	12.7 (10.2–16.9) **
**HOMA-IR**	6.4 ± 4.5 *
**NAFLD**	
No	9 (3.5)
Yes	246 (96.5)
**Body mass index**	38.7 ± 5.6 *
30–34.99 kg/m^2^	71 (27.9)
35–39.99	97 (38.0)
40 or over	87 (34.1)

* Mean ± standard deviation. ** Median (interquartile range). HDL: high-density lipoprotein; LDL: low-density lipoprotein; NAFLD: nonalcoholic fatty liver disease; CRP: C-reactive protein.

**Table 2:** Tertiles of the nutritional deficiency biomarkers in the study sample (*n* = 255).

Biomarker	Mean ± Standard Deviation
**Hemoglobin**	14.0 ± 1.5
Low tertile	12.5 ± 0.9
Intermediate tertile	14.0 ± 0.3
High tertile	15.7 ± 0.8
**Albumin**	4.6 ± 0.4
Low tertile	4.2 ± 0.2
Intermediate tertile	4.6 ± 0.1

**Table 3:** Bivariate analysis of the demographic characteristics, medical history, laboratory and clinical markers, and outcomes of interest in the study sample.

Characteristics	Albumin				Hemoglobin			
	Low Tertile (*n* = 85)	Intermediate Tertile (*n* = 85)	High Tertile (*n* = 85)	*p* Value	Low Tertile (*n* = 88)	Intermediate Tertile (*n* = 85)	High Tertile (*n* = 82)	*p* Value
Sex				0.159				<0.001
Female	25 (26.6)	37 (39.4)	32 (34.0)		7 (7.5)	19 (20.2)	68 (72.3)	
Male	60 (37.3)	48 (29.8)	53 (32.9)		81 (50.3)	66 (41.0)	14 (8.7)	
Age				0.064				0.741
18 to 29 years	14 (21.1)	25 (37.9)	27 (40.9)		23 (34.9)	23 (34.9)	20 (30.2)	
30 to 49	57 (36.5)	47 (30.1)	52 (33.3)		56 (35.9)	52 (33.3)	48 (30.8)	
50 or over	14 (42.4)	13 (39.4)	6 (18.2)		9 (27.3)	10 (30.3)	14 (42.4)	
Hypertension				0.279				0.086
No	65 (31.3)	70 (33.7)	73 (35.1)		77 (37.0)	70 (33.7)	61 (29.3)	
Yes	20 (42.6)	15 (31.9)	12 (25.5)		11 (23.4)	15 (31.9)	21 (44.7)	
Type 2 diabetes mellitus				0.627				0.099
No	78 (32.6)	80 (33.5)	81 (33.9)		84 (35.2)	82 (34.3)	73 (30.5)	
Yes	7 (43.8)	5 (31.2)	4 (25.0)		4 (25.0)	3 (18.8)	9 (56.2)	
Hypothyroidism				0.594				0.040
No	75 (33.0)	74 (32.6)	78 (34.4)		73 (32.1)	76 (33.5)	78 (34.4)	
Yes	10 (35.7)	11 (39.3)	7 (25.0)		15 (53.6)	9 (32.1)	4 (14.3)	
Smoking habit				0.253				0.689
No	66 (33.9)	60 (30.8)	69 (35.4)		69 (35.4)	66 (33.8)	60 (30.8)	
Yes	19 (31.7)	25 (41.7)	16 (26.7)		19 (31.7)	19 (31.7)	22 (36.7)	
Cholesterol				0.364				0.371
Normal	54 (36.7)	48 (32.7)	45 (30.6)		56 (38.1)	46 (31.3)	45 (30.6)	
Elevated	31 (28.7)	37 (34.3)	40 (37.0)		32 (29.6)	39 (36.1)	37 (34.3)	
Triglycerides				0.815				0.012
Normal	46 (35.1)	42 (32.1)	43 (32.8)		51 (38.9)	49 (37.4)	31 (23.7)	
Elevated	39 (31.5)	43 (34.7)	42 (33.9)		37 (29.8)	36 (29.0)	51 (41.1)	
HDL				0.025				<0.001
Normal	33 (31.1)	28 (26.4)	45 (42.5)		51 (48.1)	39 (36.8)	16 (15.1)	
Altered	52 (34.9)	57 (38.3)	40 (26.8)		37 (24.8)	46 (30.9)	66 (44.3)	
LDL				0.697				0.115
Normal	32 (35.6)	31 (34.4)	27 (30.0)		38 (42.2)	29 (32.2)	23 (25.6)	
Altered	53 (32.1)	54 (32.7)	58 (35.2)		50 (50.3)	56 (33.9)	59 (35.8)	
HOMA-IR	5.7 (3.6–8.5) *	5.3 (3.2–6.8) *	5.1 (3.4–8.3) *	0.183	4.5 (3.5–5.9) *	5.6 (3.2–7.7) *	6.2 (4.3–9.8) *	<0.001
NAFLD				0.046				0.758
No	3 (33.3)	6 (67.7)	0 (0)		4 (44.4)	3 (33.3)	2 (22.2)	
Yes	82 (33.3)	79 (32.1)	85 (34.6)		84 (34.2)	82 (33.3)	80 (32.5)	
Body mass index				<0.001				0.303
30–34.99 kg/m^2^	19 (26.8)	22 (31.0)	30 (42.2)		30 (42.3)	24 (33.8)	17 (23.9)	
35–39.99	21 (21.7)	42 (43.3)	34 (35.1)		32 (33.0)	34 (35.0)	31 (32.0)	
40 or over	45 (51.7)	21 (24.1)	21 (24.1)		26 (29.9)	27 (31.0)	34 (39.1)	
CRP				<0.001				0.377
Low tertile	18 (20.9)	33 (38.4)	35 (40.7)		30 (34.8)	28 (32.6)	28 (32.6)	
Intermediate tertile	22 (26.2)	30 (35.7)	32 (38.1)		25 (29.7)	26 (31.0)	33 (39.3)	
High tertile	45 (52.9)	22 (25.9)	18 (21.2)		33 (38.8)	31 (36.5)	21 (24.7)	

* Median (interquartile range). HDL: high-density lipoprotein; LDL: low-density lipoprotein; NAFLD: nonalcoholic fatty liver disease; CRP: C-reactive protein.

**Table 4:** Generalized linear models of the Poisson family with the log link function and robust variances to evaluate the factors associated with nutritional deficit markers in the study sample.

Characteristics	Low Albumin Tertile			Low Hemoglobin Tertile		
	aPR	95%CI	*p* Value	aPR	95%CI	*p* Value
Sex						
Female	Reference			Reference		
Male	1.86	1.26–2.73	0.002	6.94	3.37–14.32	<0.001
Age						
18 to 29 years	Reference			Not included		
30 to 49	2.02	1.24–3.28	0.004			
50 or over	2.42	1.35–4.35	0.003			
Body mass index						
30–34.99 kg/m^2^	Reference			Not included		
35–39.99	0.75	0.45–1.25	0.265			
40 or over	1.68	1.09–2.60	0.018			
Cholesterol						
Normal	Not included			Reference		
Elevated				0.71	0.52–0.97	0.034
HDL						
Normal	Reference			Not included		
Altered	1.43	1.01–2.05	0.049			
CRP						
Low tertile	Reference			Not included		
Intermediate tertile	0.98	0.59–1.65	0.952			
High tertile	1.83	1.13–2.96	0.013			

95%CI: 95% confidence interval; CRP: C-reactive protein; aPR: adjusted prevalence ratio.

## Data Availability

The datasets used to carry out this article could be available on reasonable request to the corresponding author.
